# Design and Finite Element Model of a Microfluidic Platform with Removable Electrodes for Electrochemical Analysis

**DOI:** 10.1149/2.0891902jes

**Published:** 2019

**Authors:** Daniel E. Molina, Adan Schafer Medina, Haluk Beyenal, Cornelius F. Ivory

**Affiliations:** The Gene and Linda Voiland School of Chemical Engineering and Bioengineering, Washington State University, Pullman, Washington 99163, USA

## Abstract

A microfluidic platform for hydrodynamic electrochemical analysis was developed, consisting of a poly(methyl methacrylate) chip and three removable electrodes, each housed in 1/16” OD polyether ether ketone tube which can be removed independently for polishing or replacement. The working electrode was a 100-μm diameter Pt microdisk, located flush with the upper face of a 150 μm × 20 μm × 3 cm microchannel, smaller than previously reported for these types of removable electrodes. A commercial leak-less reference electrode was utilized, and a coiled platinum wire was the counter electrode. The platform was evaluated electrochemically by oxidizing a potassium ferrocyanide solution at the working electrode, and a typical limiting current behavior was observed after running linear sweep voltammetry and chronoamperometry, with flow rates 1–6 μL/min. While microdisk channel electrodes have been simulated before using a finite difference method in an ideal 3D geometry, here we predict the limiting current using finite elements in COMSOL Multiphysics 5.3a, which allowed us to easily explore variations in the microchannel geometry that have not previously been considered in the literature. Experimental and simulated currents showed the same trend but differed by 41% in simulations of the ideal geometry, which improved when channel and electrode imperfections were included.

Miniaturized electrochemical analytical tools have been largely improved since Manz et al.^1^ first proposed the concept of a “miniaturized total chemical analysis system” (μ-TAS), which utilized a silicon-based chip for sample separation and detection. In 1993 Harrison et al.^2^ demonstrated the μ-TAS concept with a microchip that was capable of quantitative analysis in microchannels with a few nanoliters of volume. Microfluidics include systems where tiny amounts of fluids (10^−9^ to 10^−18^ L), are processed or manipulated using channels with dimensions of tens to hundreds of micrometers.^3^ Microfluidic devices have been used in various analytical operations, such as sample preparation,^4,5^ injection,^6,7^ sample handling,^8,9^ reaction,^10^ separations,^11-13^ and detection.^14^ Microfluidic analysis has been widely studied due to its inherent advantages of high sensitivity, high speed, portability, automation, and low-cost.^3,15^

Electrochemical detection of an analyte is based on a measurable electrical signal generated during redox reactions of electroactive species. Compared to other microfluidic detection methods such as fluorescence,^16^ non-fluorescent optical measurements,^17,18^ mass spectrometry,^19,20^ chemiluminescence and electrochemiluminescence,^21,22^ electrochemical detection methods offer a balance between sensitivity and selectivity and has the advantage of simple instrumentation and low electrical power consumption. The most common applications of electrochemical detection include amperometric and potentiometric detection,^23^ as well as electrochemical impedance spectroscopy (EIS).^24,25^ Amperometric detection is widely used because of its sensitivity, reliability and selectivity.^23,26^ For example, Wu et al.^27^ used a three-electrode amperometric detector integrated into a capillary electrophoresis poly(dimethylsiloxane) (PDMS) chip to measure dopamine, with an integrated platinized decoupler to isolate the interference of the separation electric field. Kang et al.^28^ performed in-channel amperometric detection of cathecol and dopamine in a capillary electrophoresis microchip, with an polyelectrolytic gel salt bridge (PGSB) to reduce the influence of the high voltage electrophoresis on the amperometric detector.

Electrochemical detection is often performed in the presence of a hydrodynamic flow. Hydrodynamic electrodes^29,30^ are those where convection is the main form of mass transfer and it includes electrodes that move through an otherwise still solution, such as the rotating disc electrode (RDE), and stationary electrodes over which a solution flows, such as the channel electrode. An advantage of these hydrodynamically swept electrodes is that the system quickly reaches steady state at a fixed flow rate, which simplifies the instrumentation and permits accurate and precise measurements at very low sample concentrations.^26,31,32^ Compared to other hydrodynamic electrodes such as the RDE, the channel electrode is more amenable to miniaturization and adaptation to on-line analysis, i.e. continuous analysis of a flowing solution, and flow injection analysis.^33^ For example, microfluidic devices have been used for flow injection analysis,^34-37^ where a small volume of electroactive analyte is injected into a carrier solution flowing through a thin-layer channel with a detection electrode. These channel electrodes come in different shapes, such as a microdisk or a microband, and as single electrodes or in electrode arrays.^30^

In electroanalysis with solid channel electrodes, the current response depends strongly on the electrode surface condition, so scratches and adsorbed materials can cause a decrease in the response of the electrode and cleaning or polishing becomes necessary to restore it.^31^ It is common for channel electrodes to be integrated into sealed microchips to achieve high reproducibility, but they cannot be replaced or easily cleaned and reused so the integrated chip must often be discarded after a few uses. Excluding disposable platforms, a common approach to accessing the electrodes for cleaning or polishing is a platform that can be disassembled into two or three pieces, with the electrodes fixed to one of these pieces. For instance, Snowden et al.,^33^ used microstereolithography (MSL) with a photoactive acrylate-based resin to fabricate a two-piece flow cell where the top piece, which contains the inlet/outlet ports as well as a 3 mm wide by 192 or 250 μm high channel, rests on a flat base in which the electrodes are embedded; and with the whole assembly held in place by a cotton thread.^33^ Sansuk et al.^37^ and Channon et al.^34^ used a similar design but with 25 μm high channels for the detection of dopamine in the former case, and 22.5 μm high channels for the detection of hydrazine in the latter case.

Adesokan et al.^38^ used a two-piece substrate where a removable cartridge-like section containing vapor-deposited gold electrodes fit into a chamber that housed the inlet and outlet ports as well as the electrical contacts, and against which a 1 mm high flow channel is formed. They tested their system by running cyclic voltammetry on ferricyanide solutions at different flow rates ranging from 50 to 250 μL/min. In a different approach, Erkal et al.^39^ used removable electrodes on a single-piece chip, where the working and pseudo-reference electrodes were epoxy-sealed together in a single 1/8” OD polyether ether ketone (PEEK) fitting and screwed directly into a 3D-printed chip with a square 500 μm × 500 μm channel. This two-electrode configuration, using a pseudo-reference/counter electrode, was utilized for the detection of nitrous oxide.

In order to characterize electrochemical analysis platforms with channel electrodes and predict the behavior of faradaic currents with changes in the flow, the concentration of reactant or variations in other key variables, extensive simulation work has been performed on this subject by a variety of researchers as previously reviewed.^40^ In particular, COMSOL Multiphysics has been used to simulate mass transfer-limited currents on single and dual microband electrodes in 2D.^33,38,41-46^ Microdisk channel electrodes have been modeled using finite differences in 3D^47-49^ using an ideal model space with perfectly rectangular channel and flush electrode, and no radial edge effects. Although 3D simulations are challenging due to the resources in RAM, storage memory and computational speed they demand, 3D calculations are required in this work because channel disk electrodes are, by their nature, non-uniformly accessible^50^ along the axial (x) and lateral (y) dimensions. That is, the mass flux profile near the electrode surface varies differently in each dimension due to the axial flow, the electrode reaction on the transverse surface and the effect of microscale confinement along the lateral and transverse axes.

In the system presented here, we use microliter per minute flows in a high aspect ratio microchannel to enhance mass transfer to the surface of a solid microdisk working electrode. The function of this working electrode is to reduce or oxidize an analyte of interest in the flowing solution, which produces an electrical current that can be measured and related to the analyte concentration and solution flow rate. This microfluidic platform consists of three removable electrodes: a platinum working electrode (WE), an Ag∣AgCl leak-less reference electrode (RE) and platinum counter electrode (CE) that closes the electrical circuit. The three electrodes are independently installed in a poly(methyl methacrylate) (PMMA) microfluidic chip with a hot-embossed 150 μm × 20 μm × 3 cm microchannel. This electrochemical platform was tested using a 5 mM potassium ferrocyanide solution by applying linear sweep voltammetry and chronoamperometry at flow rates ranging from 1 to 6 μL/min and oxidizing potentials that go up to 1 V_Ag/AgCl_. The oxidation reaction of ferrocyanide ions Fe(CN)_6_^−4^ at an anodic WE is:^30^

[1] $$Fe(CN{)}_{6}^{-4}⇌Fe(CN{)}_{6}^{-3}+e^{-}.$$

Finally, the magnitude of the mass-transfer limited current at different flow rates was estimated utilizing the finite element method in 3D using COMSOL Multiphysics 5.3a and compared with the experimentally measured current. In this software, complex channel and electrode geometries can be readily implemented to better reflect the actual geometry of the system being studied, such as channel deformation, electrode recess or radial diffusion edge effects. We introduced geometrical imperfections in the model based on channel profilometry and on scanning electron microscopy (SEM) on the electrode tip, obtaining predicted currents that are closer to the experimental data.

## Experimental and Simulation Methods

### Chemical and materials.

Potassium chloride was purchased from JT Baker (Center Valley, PA). Potassium hexacyanoferrate(II) trihydrate (K_4_Fe(CN)_6_ · 3H_2_O) (CAS No. 14459-95-1) reagent grade, was obtained from Sigma-Aldrich (St. Louis, MO). SU-8 2010 was purchased from Microchem Corp. (Westborough, MA). AZ400K was purchased from Capital Scientific (CAS No. 20786-60-1). CE-5 chromium mask etchant was purchased from Transene Company Inc. (Danvers, MA). Mylar masks and chrome masks were purchased from Advanced Reproductions (North Andover, MA) and Nanofilm (Valley View, OH), respectively. Polyetherimide (PEI) substrates (Catalog No. 8685K42) and UV transparent PMMA substrates (extruded sheets 1/8” thick, catalogue 4615T95, and 5/16” thick, catalogue 4615T46) were purchased from McMaster-Carr (Elmhurst, IL, USA). 1/8” OD × 1/16” ID and 1/16” OD × 0.005” ID PEEK tubing was obtained from IDEX Health & Science (Catalog No. 1534 and 1535, Oak Harbor, WA). Hydrophobic fluoropore filter 0.2 μm (Part No: SLFG025NS) was purchased from EMD Millipore (Austin, TX). Low-viscosity epoxy resin 105 and slow hardener 206 are from West System (Bay City, Michigan). Polishing cloths (Trident, item 40–7518) are from Buehler (Lake Bluff, IL). Polishing compounds 6 μm monocrystalline diamond (part 5DC6M), and 0.25 μm (Metadi II, part 40–62410) are from Hudson (Cleveland, OH) and Buehler, respectively. 1 and 0.05 μm alumina polishing suspensions are from Buehler. Micro-Mesh cushioned polishing sheets (2400–12000 grit) are by Micro-Surface Finishing Products Inc. (Wilton, IA). TC grade platinum wire (99.99% min) for microelectrodes was purchased from California Fine Wire Co. (Grover Beach, CA).

### Chip and electrode fabrication.

The microchip is comprised of two rectangular pieces of PMMA bonded together and was fabricated using previously published methods^15^ with some modification. The desired pattern, in this case a straight channel with 150 μm nominal width, was printed onto a mylar mask and then transferred onto an AZ1518 chrome photomask by exposure to near UV light at a wavelength of 365 nm for 30 seconds using a Hybralign Series 500 mask aligner (Optical Associates, San Jose, CA). After UV exposure, the chrome mask was developed in a 3:1 (v:v) water:AZ 400K solution for 10 seconds and etched in CE-5 chromium mask etchant until all exposed parts of the mask are transparent. The chrome mask pattern was then transferred onto a polyetherimide (PEI) substrate coated with SU-8 2010 via photolithography, to obtain a positive mold. The structure height was measured to be ~20 μm using a profilometer (Bruker DektakXT, Tucson, Arizona). The PMMA is cut into 2”×3” coupons from a 24”×48” sheet by table saw and CNC mill. Using the positive mold, a 1/8”-thick PMMA coupon was hot embossed at 110°C and an applied force of 0.24 tons for 1 min using an Atlas™ 15T Manual Hydraulic Hot-Press (Specac Inc., UK).

**Figure 1.**
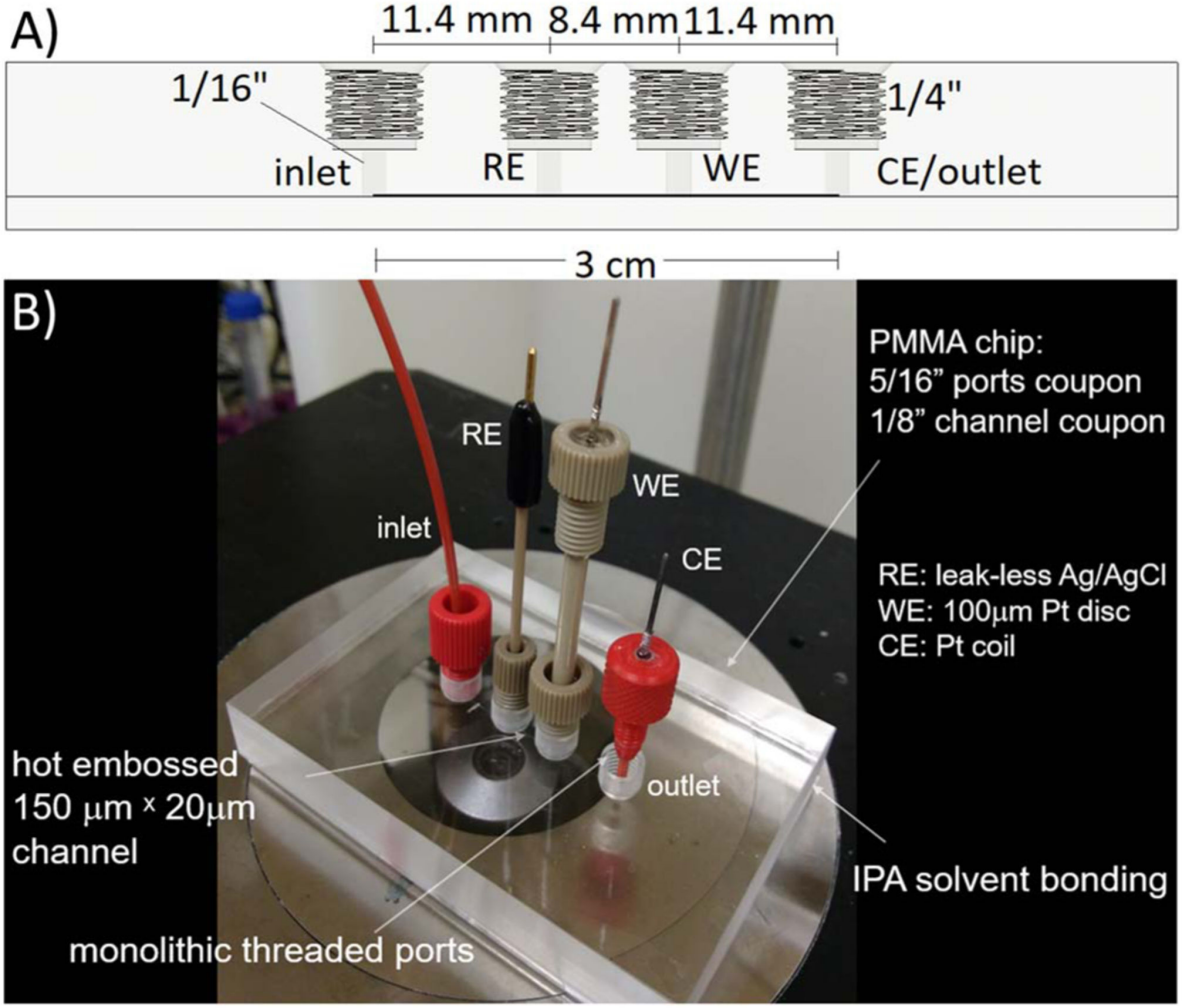
Microfluidic platform (A) side section diagram and (B) set up photo. All channel ports are 1/16” and accept $\frac{1}{4}$-28 UNF threaded fittings and ferrules for sealing. All electrode housings and inlet tubes are 1/16” OD. The chip is mounted on an inverted microscope stage.

The top PMMA coupon is 5/16” thick and has four 1/16” diameter holes that are used as access ports for the inlet, RE, WE and CE/outlet. Port holes and $\frac{1}{4}$-28 UNF threads were milled with a Nomad Carbide3D computer numerical control (CNC) machine (Torrance, CA). 1/4” PEEK fittings and plastic ferrules for 1/16” tubing are used to hold the electrode or inlet tube in place and seal it non-permanently. The top PMMA coupon was designed using Autodesk Fusion 360 software (San Francisco, CA) which also generates the G-code for the milling operations. After milling, the coupon is annealed in an oven at 100°C for 3 hours to relieve stresses created during the milling process. The imprinted PMMA coupon was aligned manually with the top coupon, and then held together with four clamps. It was bonded using a modified thermally-activated isopropyl alcohol (IPA) solvent bonding technique^51,52^ at 65°C in an oven (Lab Companion 300C, Daejeon, Korea) for 30 min. A schematic diagram of the chip is shown on Figure 1A, and a picture of the finished chip with installed electrodes is shown on Figure 1B.

To check for deformations in the hot-embossed channel, profilometry was also performed on an open channel before the bonding step. This profile was later used as a more realistic approximation of the microchannel geometry for simulation purposes.

**Figure 2.**
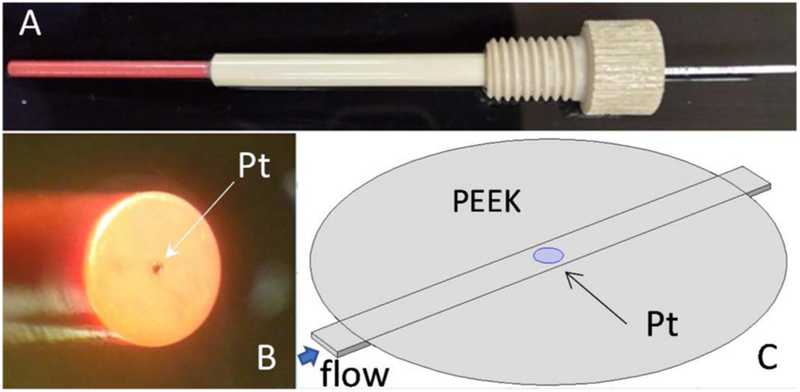
(A) Side view of the working electrode (WE) with red 1/16” and beige 1/8” PEEK tube body. (B) WE tip showing 100 μm solid microdisk electrode at the center of the 1/16” PEEK tube face. (C) Diagram of the installed electrode showing the large circular PEEK tube ideally flush against the channel’s upper surface with the small Pt electrode disk in the center. Drawing is from the COMSOL Multiphysics software.

The WE, based on modification of a design by Luther et al.,^53^ is a 100 μm diameter Pt disk. It is constructed using 100 μm OD Pt wire, sealed with epoxy inside a 1/16” OD × 0.005” ID PEEK tube. First, about 6 cm of the PEEK tubing is cut using Clean-Cut90™ tubing cutter from Microsolv (Eatontown, NJ) to achieve a cut as perpendicular as possible. The PEEK tubing is shipped by the vendor coiled on a spindle, so a thermal straightening step was included in our procedure which involved putting the tubing inside a glass tube with about 1.62 mm ID, from an old rotameter, to keep it straight inside an oven at 177°C for one hour. Then, about 7 cm of Pt wire is inserted in the tubing, leaving about 1 cm to provide an external connection. The sealing epoxy has a low viscosity and is heated to 35°C when sealing the Pt wire in the PEEK tubing; a disposable syringe with a micropipette tip is used to force as much epoxy as possible into the annulus between the wire and the PEEK lumen. We leave about 0.5 cm of the wire exposed, when performing this sealing step, and then pull in the wire from the opposite end to ensure a better epoxy seal. The electrode is then placed in our oven at 45°C for 6 hours to accelerate the curing step. Once sealed, and cured, an outer housing using 1/8” OD-1/16” ID PEEK tubing is added that covers the top 2/3 of the electrode’s length, sealed with a small bead of epoxy around the bottom. This reinforcement reduces flexing of the 1/16” body during installation, removal and polishing of the electrode. The top end of the electrode is comprised of a PEEK fitting for 1/8” tubing, where the Pt wire is tightly coiled onto a tin-coated copper pin that is epoxied to the fitting and serves as the external electrical connection point. Once the electrode is finished, about 1 mm of the tip is cut using the same straight cutter to reveal a Pt disk, sealed with epoxy on the PEEK electrode face as shown in Fig. 2B.

To polish the WE to a mirror finish so that it sits flush with the upper surface of the channel, successive one-minute polishing steps were applied using first 600 grit sand paper and then successive 2400-4000-6000-12000 grit cushioned polishing sheets. To keep the electrode perpendicular to the polishing surface, the electrode is inserted in a 1/16” diameter hole that was drilled in a PMMA block, and then moved in a figure-of-eight motion to ensure uniform polishing. The final surface finish was achieved by using polishing diamond compounds, grade 6 μm and 0.25 μm, and alumina polishing suspensions, grade 1 μm and 0.05 μm. Before each experimental run, the electrode is sonicated in 7 M HCl solution for 5 minutes to remove any deposits left from the polishing process, and the mirror finish is determined with optical microscope Olympus CK-2 (Tokyo, Japan) at 4X and 10X magnification. When installed in our microfluidic chip, the round tip of the electrode is butted flush against the microchannel and aligned in such a way that the microdisk electrode is close to the center of the channel. Figure 2 shows different views of the WE. Prior to any experimental run, if an existing electrode had been used before, it is polished starting with the 0.05 μm grade alumina suspension step if it’s not severely scratched, or with the 6 μm grade suspension if it is.

Figure 1B shows a picture of the installed electrodes. The RE is a leak-less Ag∣AgCl LF-1.6-58 electrode from Innovative Instruments (Tampa, FL), with a 1/16” OD PEEK housing. The CE was constructed with a 220 μm OD Pt wire, epoxy-sealed in inside a 1/16” OD × 250 μm ID PEEK tube housing, with 0.5 cm of the wire being coiled to an outer diameter slightly smaller than 1/16” to fit in a reservoir located below the outlet port and to permit outflow of ferrocyanide solution from the microchip. The surface area of the coiled CE is about 1.6 mm^2^, compared to 7.85 · 10^−3^ mm^2^ for the WE. The top end of the CE electrode is comprised of a coned PEEK fitting for 1/16” tubing, where the Pt wire is tightly coiled onto a tin-coated copper pin that is epoxied to the fitting and serves as the electrical connection to the electrode.

To ascertain the electrode tip profile shape beyond what was observable under an optical microscope after polishing and sonication, scanning electrode microscopy (SEM) was performed. A 1-mm piece of a used electrode tip was cut, then mounted on an adhesive stub and visualized with a FEI Quanta 200F SEM (Hillsboro, OR) at 20 kV without any coating. The SEM image is included in the Supplementary Material. Deviation from the ideal flush geometry was approximated with a recess of 5 and 10 μm, which is later included in the simulation model and shown in Figure 8.

### Experimental setup.

A three-electrode system was used as shown in Fig. 1B. All the electrodes, except for the CE port through which the solution exits the microchannel, were mounted onto the microchip using flangeless fittings (Idex Health & Science, WA) so that they can be easily installed and removed for polishing or replacement.

The electrolyte solution was continuously pumped into the flow channel via a pressurized flow well and flow sensor in feedback loop control mode (MFCS-EZ, Fluigen Inc., Lowell, MA). The flow well is pressurized with nitrogen and was connected to 1/32” ID tubing using a finger-tight PEEK fitting (Upchurch Scientific, Oak Harbor, WA). A microfluidic S flow sensor unit (Fluigent Inc., Lowell, MA) placed upstream of the microchip inlet was used to measure flow rates in the range 1–6 μL/min, with the flow rate set using Fluigen’s MAESFLOW software 3.3.1. The flowmeter calibration was checked by replacing the pressurized flow well with a PHD 2000 syringe pump (Harvard Apparatus, Cambridge, MA) and setting the pump at fixed flow rates between 1–6 μL/min with the ferrocyanide solution. The flow well was covered with black tape to block exposure of the ferrocyanide solution to light. Before starting the experiment, the microchannel was cleaned by rinsing with 1 M HCl, 18 MΩ · cm deionized water (Barnstead Nanopure Infinity, Dubuque, IA), and then 0.5M KCl background electrolyte solution. The potential on the WE was controlled by a Series G 300 Gamry potentiostat (Warminster, PA). The same has been calibrated using the manufacturer’s UDC4 calibration board. After a set of voltammetry runs or chronoamperometry, all the electrodes are removed and rinsed with deionized water. The WE is then polished as described in the previous subsection, before being reinstalled in the platform in preparation for a new run.

### Electrochemical characterization.

5 mM potassium ferrocyanide (K_4_Fe(CN)_6_ · 3H_2_O) solutions, with 0.5 M KCl as supporting electrolyte, were prepared using 18 MΩ · cm deionized water and filtered with a 0.2 μm hydrophobic fluoropore filter. This high supporting electrolyte concentration increases the conductivity of the solution and suppresses ion migration of the electroactive species. The ferrocyanide solution was prepared fresh before each experiment, and was degassed under a vacuum of 0.8 bars for 10 min to get rid of dissolved gas, with gas bubbles appearing during the first minutes and usually stopping after 9 minutes of degassing.

The trial reaction used to test the apparatus and the model was the oxidation of ferrocyanide given by Eq. 1, which is a well-proven redox probe^54^ that is stable, has fast kinetics, and is inexpensive. The electrochemical platform was characterized 1) using linear sweep voltammetry (LSV) at various hydrodynamic flow rates, i.e., 1–6 μL/min, at a potential scan rate of 5 mV/s between 0.1 - 0.8 V_Ag/AgCl_, and 2) chronoamperometry at 1 V_Ag/AgCl_ with step changes in flow rate from 1–6 μL/min. In the first experiment we will determine if typical LSV values for hydrodynamic microelectrodes are obtained using this platform and the potential at which the amperometry should be run to ensure limiting currents at each flow rate for comparison with the numerical simulation.

### Finite element simulation.

#### Model and ideal geometry.

Finite element software COMSOL Multiphysics v5.3a (COMSOL AB, Sweden) was used to prepare a 3D simulation of mass and momentum transport in the microchannel and on the electrode surface. The fluid flow is modeled using the Navier-Stokes equations together with the Equation of Continuity.^33,55^

[2] $$\rho (u\cdot \nabla )u=\nabla \cdot [-pI+\mu (\nabla u+(\nabla u{)}^{T})],$$

[3] ∇⋅u=0,

where ρ is the fluid density, **u** is the vector velocity, *p* is the modified pressure, ***I*** is the 3 × 3 identity tensor, and μ is the dynamic viscosity.

The flux, ***N**_i_*, of ferrocyanide and ferricyanide is determined by the convective-diffusion equation which describes mass transport of the electroactive species:

[4] $$N_{i}=-D_{i}\nabla c_{i}+uc_{i},$$

where *D*_*i*_ is the diffusion coefficient, *c_i_* is the concentration of the *i*^th^ electroactive specie. In this equation, migration of electroactive species is not considered due to an excess of inert background electrolyte (0.5 M KCl). This highly-conductive solution also reduces the uncompensated voltage drop.^30^ The terms in Eq. 4 account for diffusion and convection, respectively. The steady state equation of conservation of mass for each specie is,

[5] $$\nabla \cdot N_{i}=0.$$

The previous set of equations in three dimensions don’t have an analytical solution and a numerical method, finite element, is used to solve them. The COMSOL simulation couples the Laminar Flow and Transport of Diluted Species physics to simultaneously model mass and momentum transport in the microchannel. Included in these physics are the Navier-Stokes equations, the Equation of Continuity and the Equation of Conservation of Mass.

**Figure 3.**
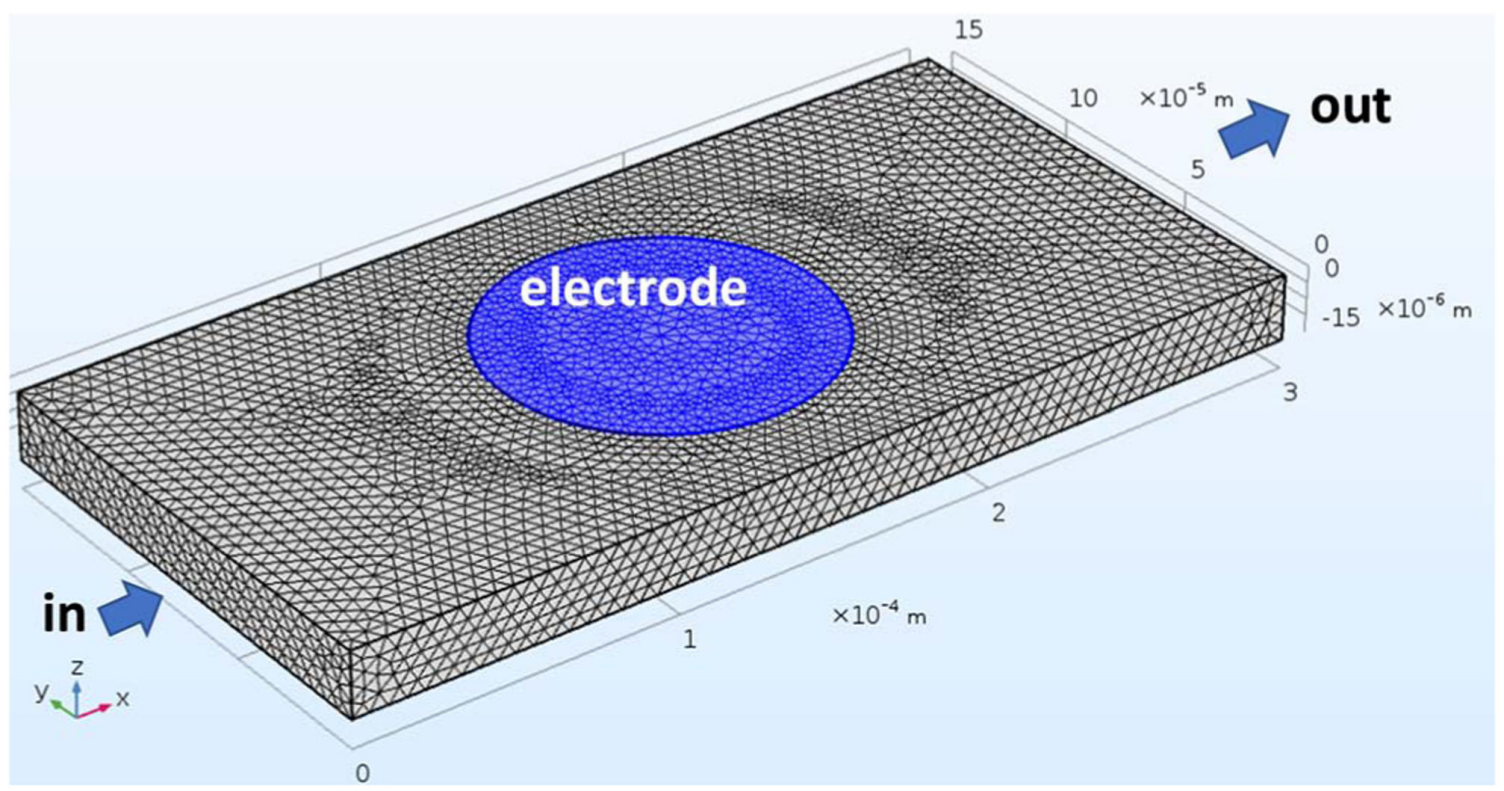
Modeled space of the 150 μm × 20 μm ideal geometry microchannel and inlaid solid microdisk electrode, diameter = 100 μm, in COMSOL Multiphysics 5.3a, for the simulation of the mass transfer-limited current. An initial coarse mesh with about 50k DOF is shown for clarity but is ultimately raised to higher mesh densities for more accurate calculation of the limiting current. A modified geometry with a denser, improved mesh is shown on Figure S6 of the Supplementary Information.

The microchannel is approximated as a 150 μm × 20 μm rectangular block with a length of 0.3 mm and with the WE centered on and flush with the top wall. A 3D model is necessary in this case as the channel disk electrode is non-uniformly accessible^50^ along the *x* and *y* axes. Figure 3 shows the geometry of the 3D ideal model used. Variations of this ideal geometry were later introduced and are described in the next subsection.

All the solid surfaces, including the electrode, are subject to the noslip boundary condition. We defined an inlet boundary condition with a fixed volumetric flow *V_f_* with values 1–6 mL/min for each run and a fully developed channel flow profile, which is a good assumption when the entry length is shorter than the distance from the last transition to the electrode. To characterize the flow, the Reynolds number in the channel is calculated with Eq.(6-7),^33^

[6] $$D_{h}=\frac{2hw}{h+w},$$

[7] $$Re=\frac{\rho u_{x}\;D_{h}}{\mu },$$

where *D_h_* is the hydraulic diameter and *Re* is the Reynolds number. Since we defined an inlet developed flow boundary condition, to facilitate convergence the outlet boundary is subject to an arbitrary pressure condition, where the relative pressure is equal to the atmospheric pressure, *p*_*atm*_, of 1.013 bar.

The inlet is subject to a Dirichlet^56^ condition where the ferrocyanide concentration is fixed at 5 mM. There is a Neumann^56^ boundary condition of zero flux for the electroactive species on all the solid surfaces except the electrode surface. Moreover, the electrode surface has a Dirichlet boundary condition where concentration of ferrocyanide is equal to zero, as the ferrocyanide oxidation reaction is fast at large potentials, so that all the ferrocyanide that can diffuse to the electrode is electrolyzed and the current is mass-transfer limited.^30^ Well-defined mass-transfer limiting curves obtained at high potentials in our experiments supports this chosen boundary condition. The boundary conditions are summarized in Table I. For the outlet there is a Danckwerts flux boundary condition^57^ that is applicable when mass transport across the boundary is dominated by convection, like in our case where the Peclet number is higher than 170.

The electrode radius, r = 49.7 ± 0.4 μm, was determined by inverted optical microscope Nikon Eclipse Ti-S (Melville, NY) using 10x magnification and which had been previously calibrated with a reticle. The ferrocyanide diffusivity has been widely reported and the value used in this simulation, D = 6.6 · 10^−6^ cm^2^/s, was obtained from the literature,^58,59^ at similar ambient conditions and background electrolyte.

The limiting current density at the electrode, *i*, is proportional to the ferrocyanide flux ***N*** at height 0, i.e. the electrode surface, according to Eq. 8:

[8] i=nF(n⋅N),

where ***n*** is the normal unitary vector at the electrode surface, *n* is the number of electrons transferred by the electrode reaction, with *n* = 1 in this case, and *F* is the Faraday constant, with *F* = 96485 C/mol. The total current at the electrode is then determined by integrating numerically the current density at each electrode mesh node, over the surface area of the electrode:

[9] $$I=\iint_{A_{e}}idxdy,$$

where *A*_*e*_ is the area of the electrode surface.

Meshing of the finite element model started with low number of nodes (10k) to a very dense mesh of about 2 million nodes, corresponding to about 12.4 million degrees of freedom (DOF). A Dell XPS 8700 desktop computer (Round Rock, TX) with an Intel i7-4790 CPU Quad Core @3.6 GHz and 32 GB of RAM running Windows 7 Professional, was used for the simulations.

#### Modified geometry with imperfections.

After running simulations with the idealized geometry show in Figure 3, geometrical imperfections in the electrode tip and the microchannel that were evidenced through SEM and profilometry were included in the model. The simulation was repeated first with a trapezoidal channel geometry, then with this channel modification plus two different recess levels in the electrode. The Supplemental Material has the profilometry and SEM data that support these modifications.

## Results and Discussion

### Electrochemical characterization of the microfluidic platform.

**Figure 4.**
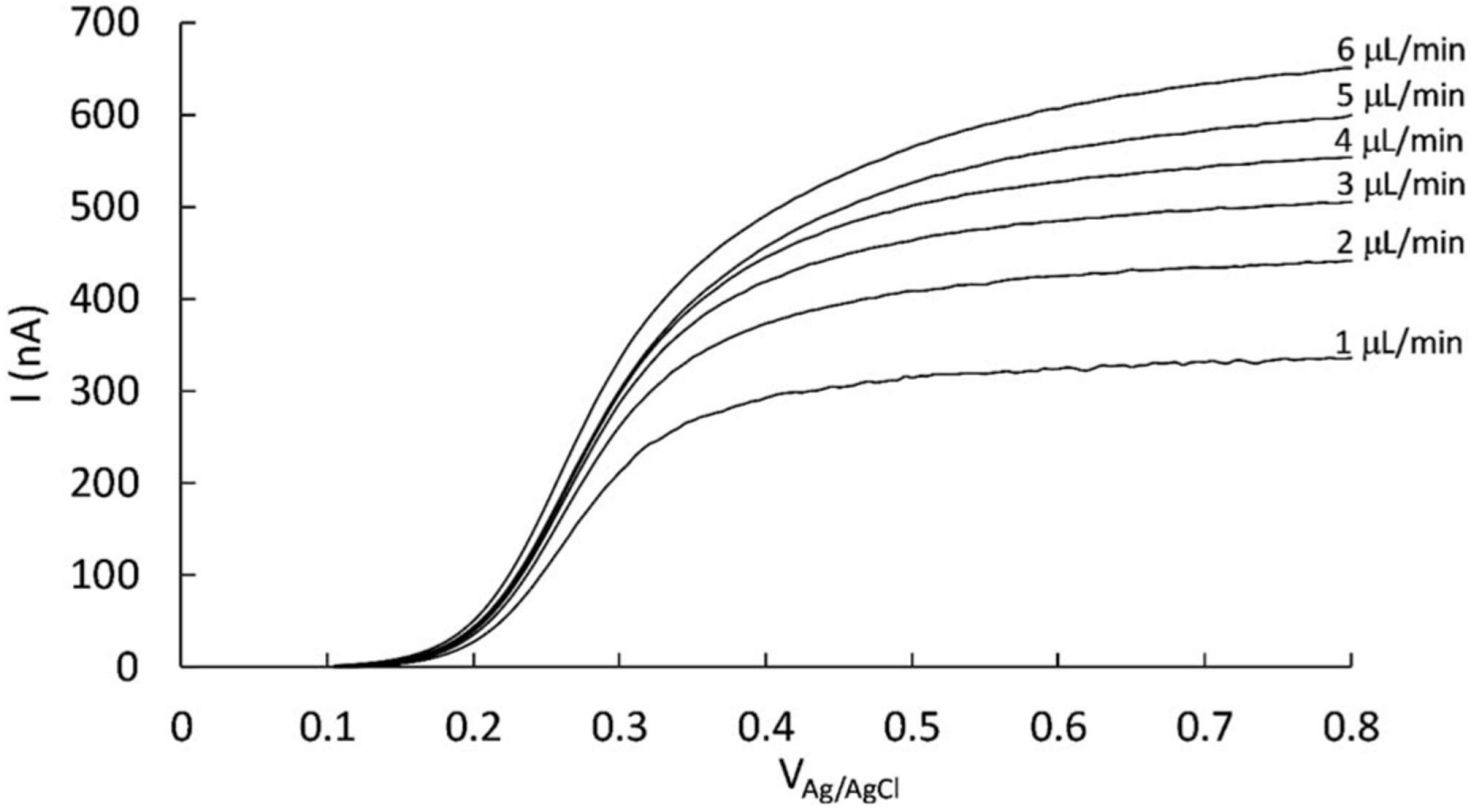
LSV for the oxidation of ferrocyanide on a microdisk electrode, showing the effect of flow rate on the limiting current. Currents increase with potential (kinetic control) and limiting currents are reached at potentials of at least 0.8 V_Ag/AgCl_, which will be used later for chronoamperometry. Flow rates = 1–6 μL/min, scan rate = 5 mV/s, solution is 5 mM potassium ferrocyanide in 0.5 M KCl, nominal electrode diameter = 100 μm.

The results of hydrodynamic LSV runs performed at different flow rates are shown in Figure 4, where ferrocyanide was oxidized at the WE. For the range of flow rates studied, 1–6 μL/min, the Reynolds number based on the inlet velocity was found to lie in the range 0.2 < Re < 1.2, ensuring laminar flow with little or no vortex formation at corners or holes. The LSV curves obtained show the typical initial potential-dependent current (kinetic control) that then turn into an asymptotic current at higher potentials. Because of the applied convection and low scan rate, we observed typical steady-state or quasi-steady state LSV curves as reported in the literature,^33,34,37^ showing no peaks and characterized by an asymptotic limiting current and half-wave potential, where the latter is the potential corresponding to half of the limiting current.^30^ The limiting current increases as we increase the flow rate, because the current is entirely mass-transfer limited rather than being partially reaction-rate limited.^48^ This is advantageous for detection purposes since it increases sensitivity by increasing the current at the WE.^30,60^

Hydrodynamic LSV, such as the one shown in Figure 4, is used in this case to determine the operating electric potential for amperometric detection.^26^ The LSV curves in that figure suggest that we can expect limiting currents when operating at a fixed potential at or above 0.8 V_Ag/AgCl_, within the range of flow rates studied. When we tested higher anodic potentials we observed a steep increase in the current at potentials above 1.1 V_Ag/AgCl_, corresponding to water electrolysis.^31^ As a result of the LSV experiments, the potentials that were used for subsequent chronoamperometry experiments are close to 1 V_Ag/AgCl_, but not higher, in order to avoid water electrolysis.

**Figure 5.**
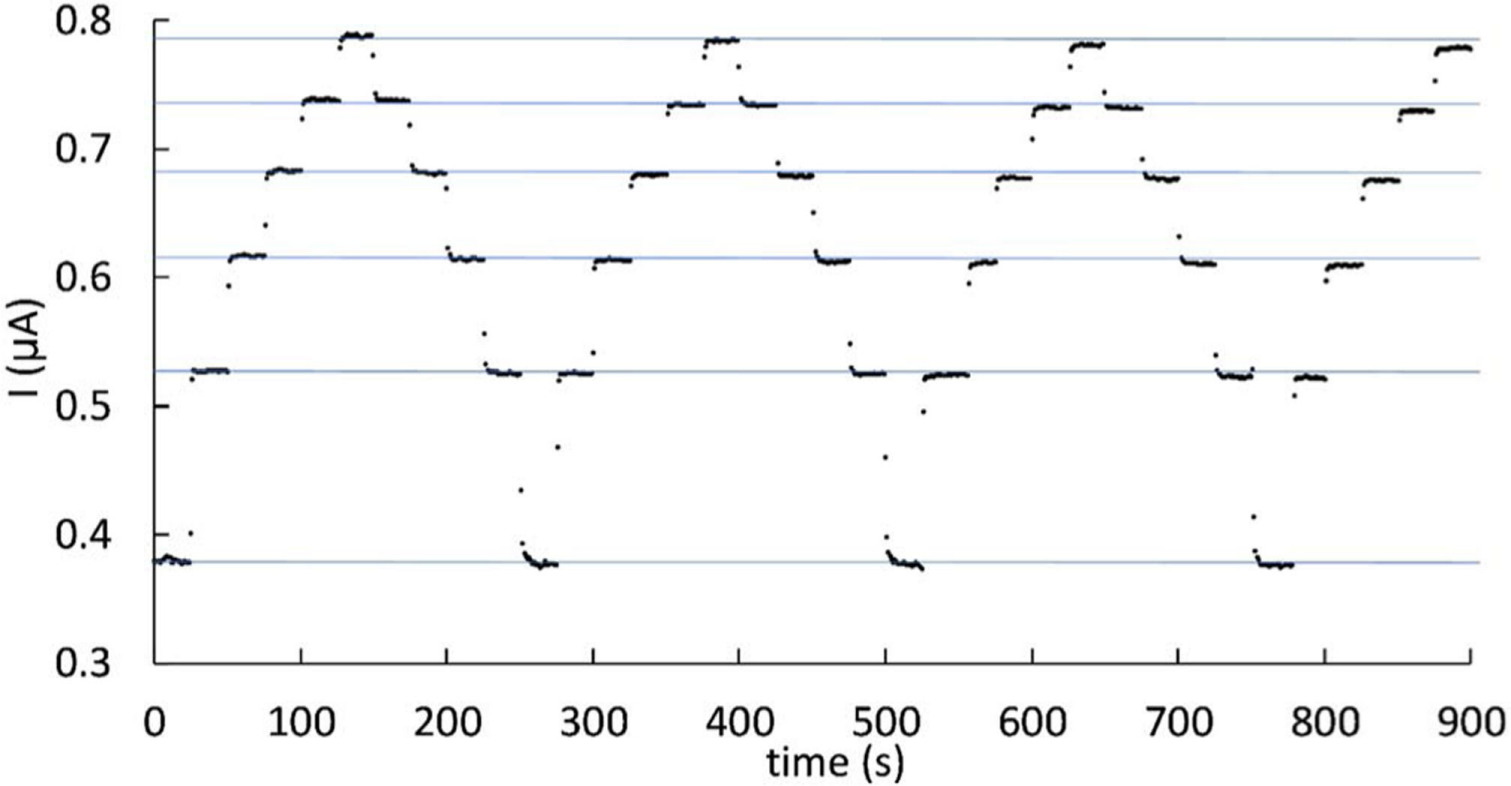
Chronoamperometry showing limiting currents at intermediate steady-states for several cycles of changes in flow rates, V_f_ = 1, 2, 3, 4, 5, 6 μL/min, for the oxidation of 5 mM ferrocyanide in 0.5M KCl in a microchannel, at a constant potential of 0.95V_Ag/AgCl_. The average limiting current within each flow rate decreased by less than 1.2% after 3.5 cycles.

To study the behavior of limiting current with changes in flow rate at a constant potential and constant inlet concentration, chronoamperometry was run at a potential of 0.95 V_Ag/AgCl_ while changing the flow rate in steps, as shown in Figure 5. The flow rate was changed by manually entering a new flow set point in the Fluigen software that controls the flow-well pressure. Once the current has stabilized after each flow rate change, the relative standard deviation of the limiting current value is less than 0.4% at each flow rate. After the first half cycle between 1–6 μL/min there’s a small decrease in the corresponding average limiting current, but this decrease is never higher than 1.2%. This small decrease could be due to a minor passivation of the WE by adsorbed ferrocyanide/ferricyanide species or a complex of these (KFe^II^[Fe^III^(CN)_6_]) on the electrode surface,^61,62^ after the solution has been flowing over it for several minutes at a relatively high applied potential.

### Finite element simulation results and comparison with experimental data.

First we check the validity of our diffusion-convection model (Eq. 4). A COMSOL simulation that considers ion migration and the effect of background electrolyte, using the complete Nernst-Plank Equation,^30^ is included in the Supplementary Information, but the resulting current at the electrode is the same within 1% as in our current diffusion-convection model, due to the excess of electrolyte employed.

#### Ideal geometry model.

**Figure 6.**
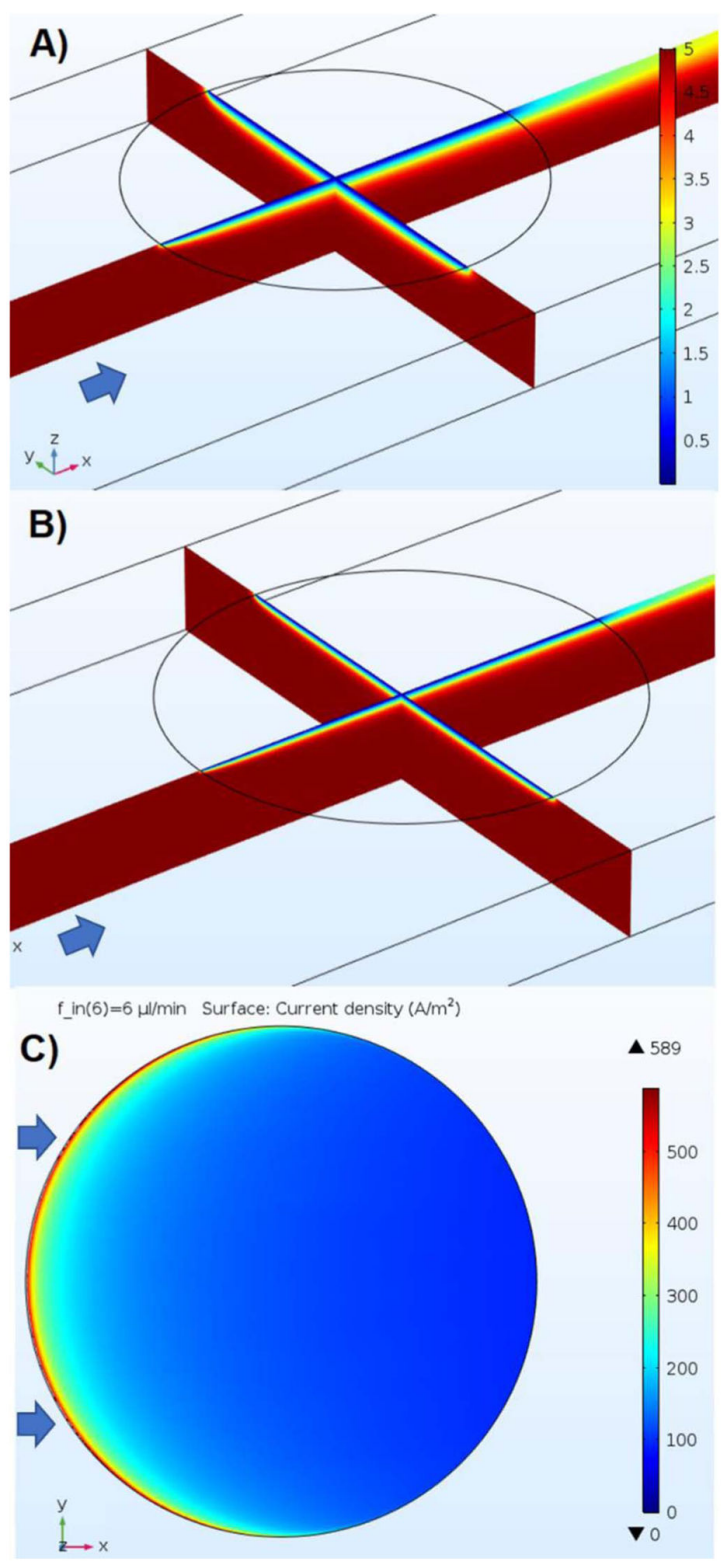
Modeled steady-state ferrocyanide concentration profiles in the solution flowing under the electrode and current density distribution on the electrode installed on the top wall of the microchannel, for mass-transfer limited oxidation of 5 mM ferrocyanide solution with (A) V_f_ = 1 μL/min, (B) V_f_ = 6 μL/min, shows that the diffusion layer thickness varies with position and flow rate due to convection over the circular electrode (C) Mass-transfer-limited current density distribution on the electrode at *V_f_* = 6 μL/min shows variation in *x* and *y* dimensions.

During the implementation of this simulation, it became evident that the results change widely with the density of the finite element mesh, i.e., with the number of nodes or degrees of freedom (DOF) of the simulation. This is confirmed in the literature, for instance, in a 3D COMSOL model of a disk and a square electrode under stagnant conditions, the mass transfer-limited current increased asymptotically when the DOF of the model was increased.^63^ The meshing of the model domain is critical to the accuracy of the simulation,^64^ and needs to be denser in the neighborhood of the electrode surface, especially at the electrode edge where the most rapid change in the concentration gradient occurs. To this end, meshing of increasing density is applied until the solution varies less than 1% in the limiting current. Meshing independency check and additional meshing information is included in the Supplementary Material. The steady-state COMSOL model for the microdisk electrode started off having about 100k DOF and was ultimately solved with little over 12 million degrees of freedom in about 3 hours for the 6 flows that were studied. Figures 6A, 6B show the simulated ferrocyanide profiles that are established adjacent to the electrode at flow rates of 1 μL/min and 6 μL/min, respectively; Figure 6C is the current density distribution on the metal electrode disk at a flow rate of 6 μL/min.

Increasing the flow rate causes an increase in the mass fluxes due to thinning of the diffusion layer as can be observed when comparing Figures 6A and 6B. These figures show how the diffusion layer thickness near the electrode varies with position in all three dimensions over the electrode, making the electrode non-uniformly accessible with respect to mass transport^47,48^ so no plane of symmetry exists that would allow an accurate simulation to be performed in 2D. The increase in analyte flux at the electrode surface is translated into an increase in current density, according to Eq. 8.

Figure 6C shows how the current density varies on the electrode surface along the direction of the flow: it is over 6 times greater at the leading edge compared to the trailing edge of the electrode, since the leading edge continually receives unreacted solution by convection, where the diffusion layer is thinner, and the mass flux of reactant is increased. Because of the electrode’s circular shape, the leading and trailing edges are also subject to lateral diffusion, so the current density also varies in the *y* direction. Apart from the lateral diffusion, our model also considers the change in current density due to variation of the velocity profile along the *y* direction. Although the microdisk is 25 mm away from each side of the channel walls, it’s close enough so that the magnitude of the velocity is smaller at the edges nearer to the channel walls. In our geometry this reduction is only ~2% for the centerline velocity and previous channel disk numerical models^47-49^ don’t take it into account, but our model can handle a disk of a size or placement that puts it arbitrarily close to the channel walls where the effect will be higher.

**Figure 7.**
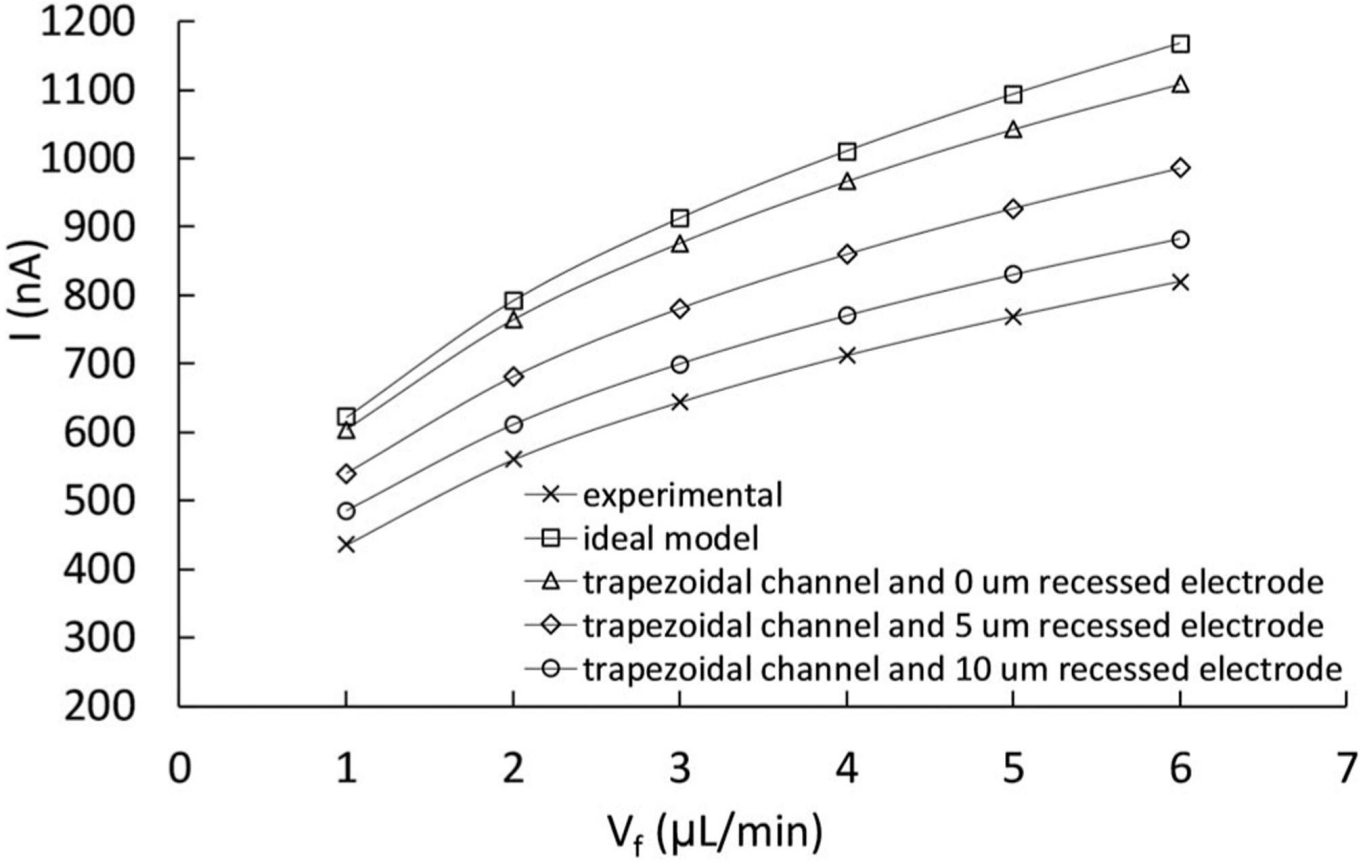
Comparison of the experimental vs predicted limiting current at different flow rates, for the oxidation of 5 mM ferrocyanide in 0.5M KCl on a 100 μm diameter WE at 1.0 V_Ag/AgCl_, installed in a microchannel. When the imperfections (trapezoidal channel and recessed electrode) are introduced to COMSOL model, the currents differ by less than 10% for the largest expected electrode recess of 10 μm, and by 20% for a 5 μm recess.

Chronoamperometry at the same flow rates and concentration as in Figure 5 was repeated on a new, freshly polished electrode at a constant potential of 1.0 V_Ag/AgCl_. These results are plotted in Figure 7, together with the predicted limiting currents calculated with COMSOL. In that figure, the simulation limiting currents are about 41% higher than the experimental data, although they follow a similar trend and the shape is similar to the curves reported in the literature for channel disk electrodes.^48,49^

#### Modified geometry with imperfections.

The difference between the predicted current by the model with ideal geometry and experimental data was significantly greater than we had anticipated so we first tested our model solution strategy against a trusted asymptotic analytical solution for the microband channel electrode, the Levich equation.^30^ The results of that comparison are given in the Supplementary Material but, basically, they show an error of less than 1.5% between the analytical solution and the simulation at a DOF ~3 million and an error of less than 1% at a DOF ~6 million.

**Figure 8.**
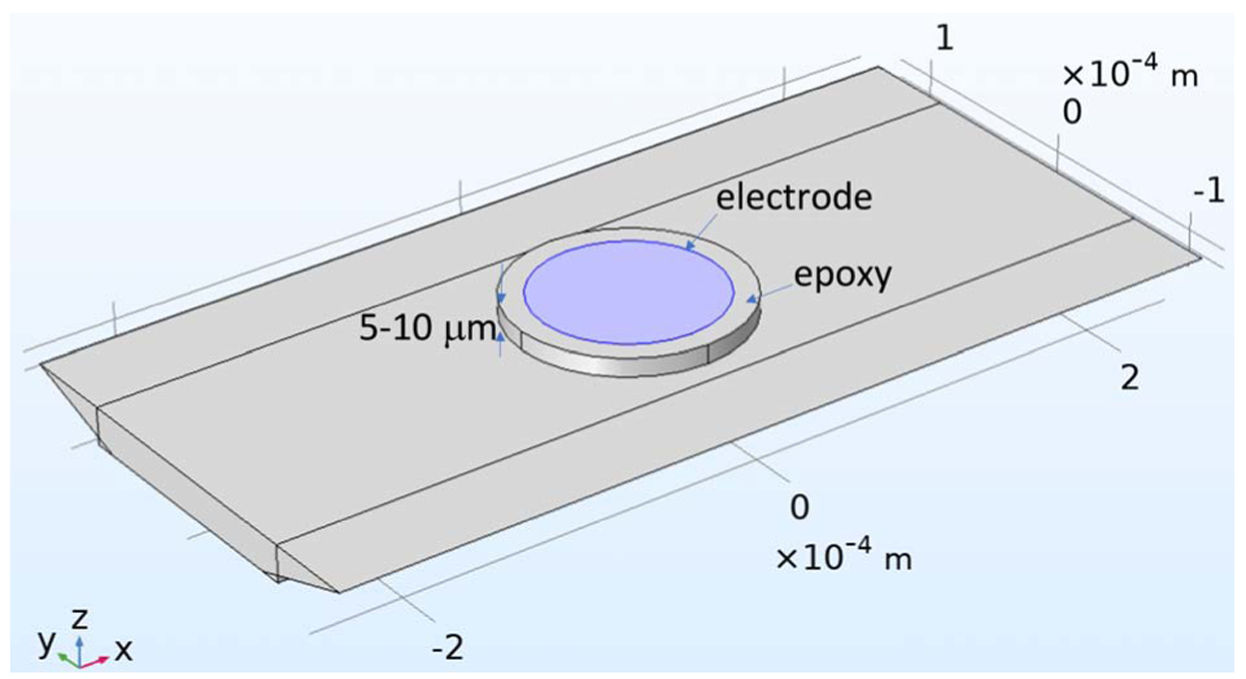
Modified model geometry incorporating deformations in the channel and the electrode. The channel is deformed into a trapezoid and the electrode sealed in epoxy is shown is recessed a maximum of 10 μ m into the PEEK housing. The meshing for this model appears in Figure S6 of the Supplementary Information.

While the discrepancy between simulation and experiment might be due to any number of deviations from the idealized model, we considered imperfections that we were able to measure or have some evidence of: 1 a geometrical variation in the channel shape, and 2 that the electrode was not flush with end of the PEEK housing but was instead recessed in the lumen of the PEEK tube. Profilometry performed on the microchannel revealed that it had a trapezoidal shape, so this was incorporated in the model geometry in COMSOL as shown in Figure 8. This deformation in the geometry primarily impacts the velocity profile adjacent to the electrode with lower velocities generating lower currents. The resulting limiting currents with this modified model geometry are included in Figure 7. In this case, the predicted currents are about 36% higher than the measured currents. This shows the channel deformation has a non-negligible impact on the limiting current, but they’re normally not considered for this type of electrochemical platform in the literature.

The SEM performed on the cut tip of a used WE revealed some recession of the electrode and sealing epoxy into the PEEK tube lumen, so that it is no longer flush with the flat tip of the PEEK housing. To show the effect of a disk electrode recess at different heights, we performed simulations with heights of 0, 5 and 10 μm; the geometry with the 10 μm recess is shown in Figure 8. The SEM image is included in the Supplementary Material. This recess reduces the flow velocity over the electrode making the diffusive layer thicker and resulting in lower currents. The roughness of the sealing epoxy might also be disrupting the laminar profile over the electrode. The limiting currents that consider geometrical imperfections are shown in Figure 7. If a recess of 5 or 10 μm is included, the predicted currents are now ~20% or ~10% higher than the experimental ones, respectively. These results suggest that deformations in the electrode position, i.e. recess, have a bigger effect on the limiting current than the channel deformations, in the ranges studied in this work.

The ability to model the mass transfer-limited currents and current densities with changes in flow rate and geometry provides insights for amperometric detection and design changes of this platform. If the enhancement that convection brings to the limiting currents can be accurately predicted, it gives us confidence that the model can be used to explore variations in design that will eliminate bottlenecks in mass transfer and lead to a more efficient platform. If conversion efficiency is not important to the detection process, smaller disc electrodes or even narrow band electrodes with more of its total area in the leading edge can be used. If higher conversion rates are required. the present design can be used at lower velocities to produce currents that are enhanced by convection. Ensuring that the working electrode is flush can significantly increase the limiting currents and, hence, the conversion rates or sensitivity of the platform.

For the microfluidic platform to have a predictable response it should have a fully developed laminar flow profile over the electrode, so we check the entry length of our microchannel. Using an empirical relation for trapezoidal microchannels at low Reynolds numbers,^65^ we estimate this to be about 19 mm for the highest flow of 6 mL/min, which is much shorter than the actual ~740 μm available from the last channel transition to the electrode, i.e. the microchannel zone under the PEEK+Epoxy substrate of the electrode tip shown on Figure 2C.

## Concluding Remarks

A microfluidic platform with three removable electrodes: microdisk WE, RE and CE, was demonstrated for a microchannel of nominal size 150 μm × 20 μm × 3 cm and volume of 90 nL, with a height and channel volume much smaller than previously reported for this type of removable electrodes. The electrodes have the advantage that they are cheap, simple to fabricate and can be removed for polishing or replacement. The small channel height allowed the use of flow rates below 6 μL/min, while still maintaining a velocity profile that enabled enhanced mass transfer of the ferrocyanide analyte to the working electrode surface where it is oxidized. Laminar flow with low Reynolds numbers close to 1 was maintained throughout.

This platform was tested utilizing hydrodynamic LSV and chronoamperometry, where mass-limited currents increase with increasing flow rate. The limiting currents at a given flow were stable, and after repeated cycles of flow rate changes between 1–6 μL/min, the corresponding currents decreased by less than 1.2% after 3.5 cycles.

The microdisk channel electrode is non-uniformly accessible and had been modeled numerically in 3D using finite differences with an ideal model space. In this work we predicted the mass transfer-limited currents using a finite element method in COMSOL Multiphysics 5.3a. Reasonable agreement with experimental data was obtained once more realistic geometrical features of the actual channel and electrode were considered. Removable channel disk electrodes are relatively easy to manufacture, and this simulation demonstrated that modeling more complicated geometries in 3D is feasible in COMSOL, using moderate computing power and time. This frees the modeler from ideal geometries and other common simplifications in 2D such as the Leveque approximation for the flow velocity or discarding radial and axial diffusion, with a modest computational cost.

Due to the impact of deformations on the limiting currents, in the future we expect to refine the profile determination of the closed channel and electrode surface to improve our model and apply it to electrochemical detection.

## Supplementary Material

text

## Figures and Tables

**Table I.** Boundary conditions used in the simulation of the mass-transfer limited current at different flow rates in a microdisk channel electrode.

Boundary	Navier-Stokes boundary condition	Convection-diffusion boundary condition
solid surfaces	*u*_*x*_ = 0, no-slip	***n*** · ***N*** = 0, insulating
electrode surface	*u*_*x*_ = 0, no-slip	*c_ferrocyanide_* = 0
inlet	*V_f_* = 1 – 6 μL/min, fully developed flow	*c_ferrocyanide_* = *c*_0,*ferrocyanide*_
outlet	*p* = *p_atm_*	***n*** · (−*D*∇*c_i_*) = 0

Where **N** is the inward ferrocyanide flux, c_0,ferrocyanide_ is the entering ferrocyanide concentration (5 mM), **n** is the vector normal to a particular boundary
