# Supplementary material for: Design and Finite Element Model of a Microfluidic Platform with Removable Electrodes for Electrochemical Analysis
Source: J Electrochem Soc. Author manuscript; Available in PMC 2019 Jul 24. (PMC6656400; doi:10.1149/2.0891902jes)
Supplement: text [file NIHMS1034102-supplement-text.pdf]

Design and Finite Elements Model of a Microfluidic Platform with Removable Electrodes for Electrochemical Analysis

Daniel E. Molina, Adan Schafer Medina, Haluk Beyenal and Cornelius F. Ivory*

Affiliations (all authors): The Gene and Linda Voiland School of Chemical Engineering and Bioengineering, Washington State University, Pullman, WA 99163, U.S.A.

*Corresponding Author: Cornelius F. Ivory

The Gene and Linda Voiland School of Chemical Engineering and Bioengineering, Washington State University, P.O. Box 646515, Pullman, WA 99164-6515 U.S.A.

Email: cfivory@wsu.edu

Fax: (509) 335-4806

Supplementary Material

This Supplementary Material includes profilometry data of the channel and an SEM image which were used to alter the geometry of the rectangular channel model used in the simulation from its ideal, rectangular shape to a more realistic shape. Also included are the meshing sequence and mesh properties used in the COMSOL simulation for the ideal and modified channel geometries. The simulation of a 3D band channel electrode using COMSOL is compared against the analytical Levich equation to validate our custom meshing strategy and 3D finite element approach. Lastly, simulations using the complete Nernst-Plank equation are included, which give similar results to the ones presented in the main paper using the simple diffusion-convection.

1. Profilometry

Profilometry was performed on a typical unbonded PMMA hot-embossed substrate to determine the channel's profile and then modify the COMSOL model geometry to approximate the actual channel shape. A Bruker DektakXT profilometer was used (Tucson, Arizona) and results are shown on Figure S1.

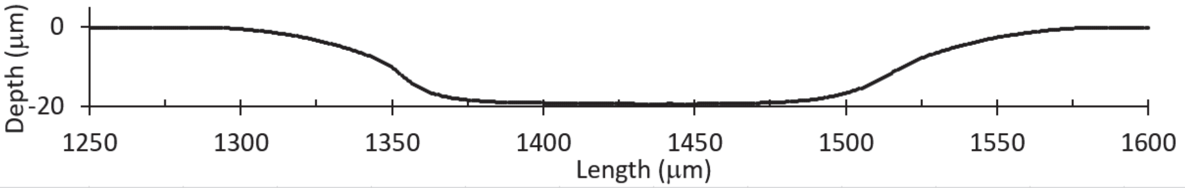

Figure S1. Channel profile along the lateral axis of a hot-embossed microchannel before bonding; this profile was later used to modify the model geometry in COMSOL. The hot-embossing produces the desired channel height along the midsection, but the walls are not vertical, and they have a steep rising curve at about the ideal width but then rise with a lower slope and open to a total width of about 250 µm.

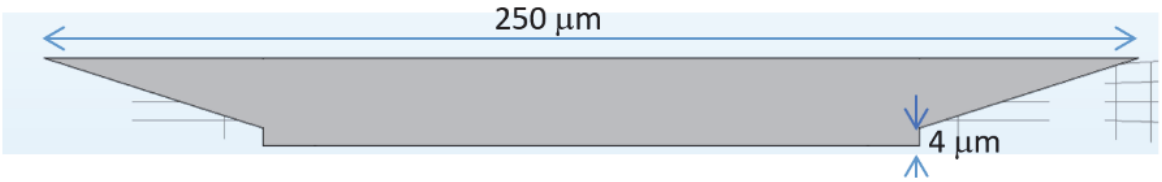

Figure S2. Modified channel geometry in COMSOL that approximates the actual channel profile. The 150 μm channel expands to a total width of 250 μm at the top and is approximated with straight lines for simplicity. This change in geometry impacts the velocity profile, and in turn the limiting current at the electrode that sits on top of the channel.

The ideal 150 μm × 20 μm rectangular channel geometry was modified to the one shown in Figure S2 for the COMSOL model. The change in geometry lowers the velocity near the electrode, so the mass-transfer limited current is also lower due to reduced convection. This is demonstrated in Figure 7 of the main article. In addition to this channel modification, a recess in the electrode tip is discussed below.

2. SEM imaging of electrode tip

To prepare a more accurate description of the electrode interface on the upper surface of the microchannel, Scanning Electron Microscopy (SEM) was performed using a FEI Quanta 200F SEM (Hillsboro, OR) on a thin section of a used electrode that had been polished at least 5 times between runs in our microfluidic platform.

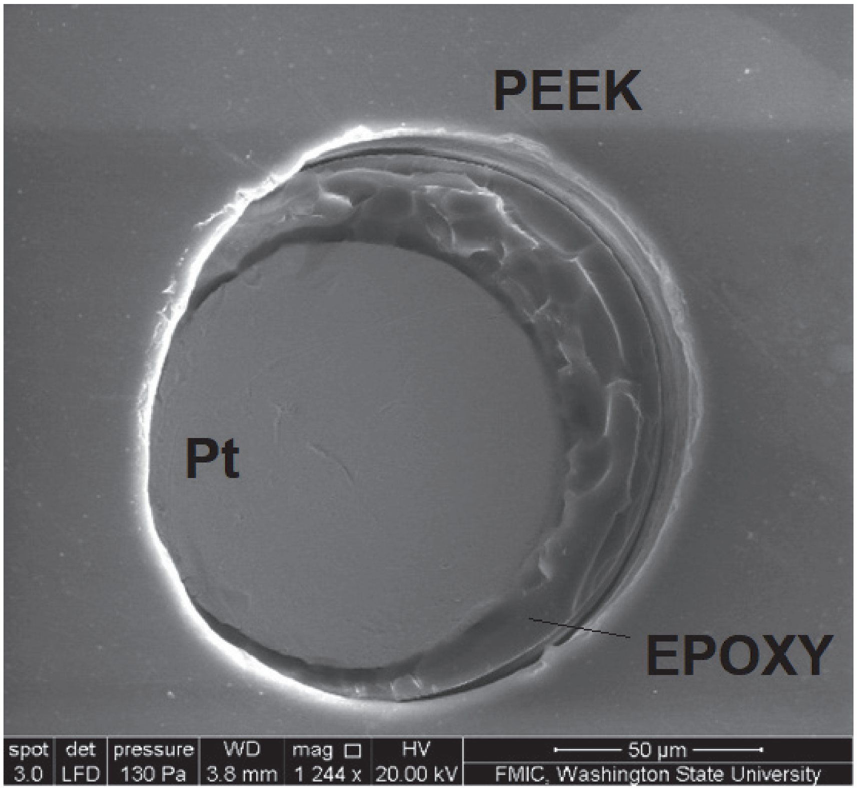

Figure S3. SEM image of recessed electrode in the PEEK tube housing. The sealing epoxy is rougher than expected and the 100 μm diameter electrode is recessed relative to the surrounding PEEK surface.

From the image of the electrode tip taken by SEM and comparing with other electrodes we fabricated, we estimate that the depth is no more than 10 μm, so we used that value to construct the modified geometry of the recessed electrode that appears in Figure S6. This recess might be due by repeated polishing with diamond paste and alumina suspension, since we have 3 dissimilar materials on the electrode tip. The recess reduces the velocity around the electrode and the impact on the limiting current is shown on Figure 7 of the main article. We also include a simulation with an intermediate recess of 5 um to better show the trend in the limiting current with recess height. The incorporation of a recess in the model, together with the channel deformation, can explain part of the difference between simulated and experimentally measured limiting current.

3. Model mesh and iterations

Meshing has a significant impact on the accuracy of the electric current that is predicted in COMSOL.[1, 2] In our case, we want to be able to use a denser mesh on the electrode surface and around the electrode-insulator edge where the concentration gradient is greatest. To this end, we implement a user-controlled type of meshing, as opposed to the physics-controlled meshing that COMSOL provides by default. The meshing parameters for the ideal channel case are summarized on Table S1.

We start with a coarse mesh and work our way up to higher mesh densities until the resulting current converges to a stable value changing less than 1% when we increase the mesh by at least 1 million nodes. The meshing sequence properties used for the same ideal channel case at the different meshing densities are also summarized in Table S1. Figure S5 shows the convergence of the resulting limiting current at increasing mesh densities for the same channel electrode. Beyond ~600k mesh vertices or nodes, there's no significant increase in the limiting current at any of the flowrates used. This analysis can be done similarly using the degrees of freedom of the model at a given mesh density, since the number of nodes or element vertices correlates directly with the number of degrees of freedom (DOF), with DOF = # nodes $\times$ # dependent variables.

After the ideal channel model simulations were completed, the model with the trapezoidal geometry in Figure S2 was used to simulate the limiting currents. A recessed electrode was added to the model geometry of Figure S2 to produce the modified geometry in Figure S6, where the mesh is also included. The electrode border mesh density is high, in addition to the meshed areas on the top outer edges of the channel that have become very thin. The meshing sequence properties used for the modified channel cases, trapezoidal channel and trapezoidal channel+recessed electrode, at high meshing densities is summarized in Table S2. The incorporation of these modified geometries results in limiting currents that are closer to the experimentally measured ones, as shown in Figure 7 of the main article.

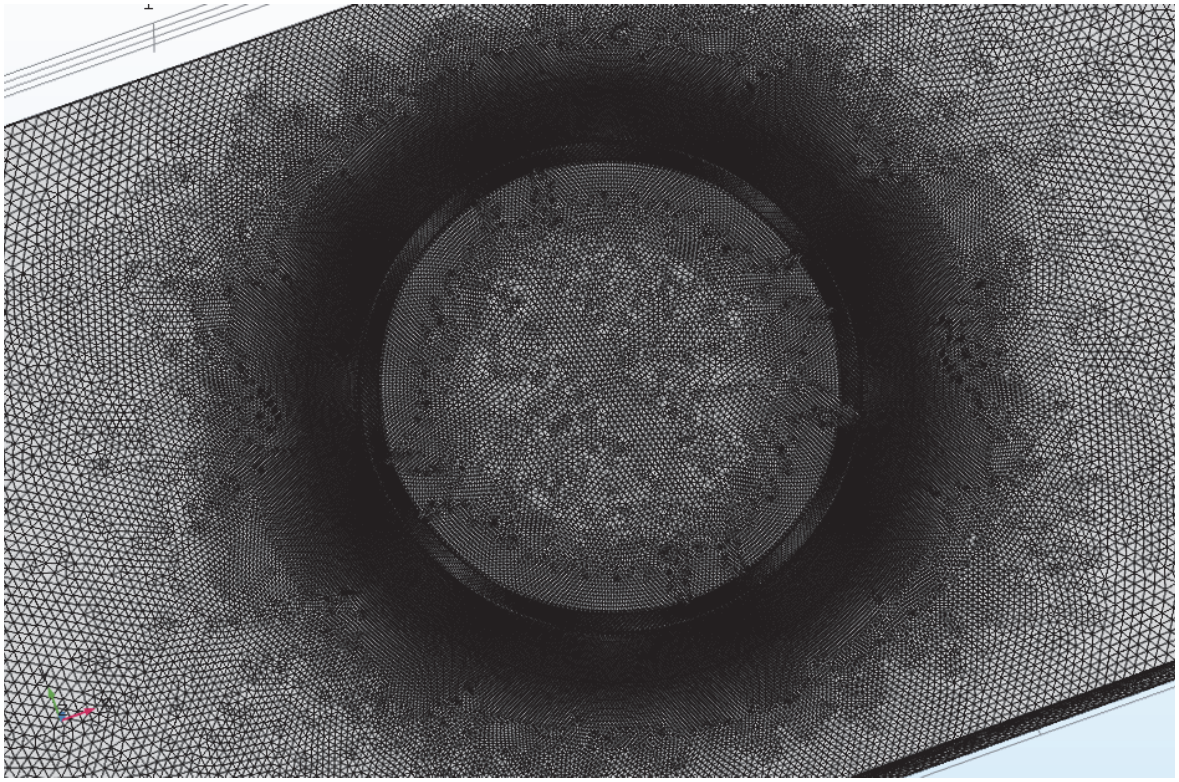

Figure S4. COMSOL model meshing over the inlaid electrode at 618k nodes. The mesh denser at the electrode-PEEK interface where the concentration gradient change is highest.

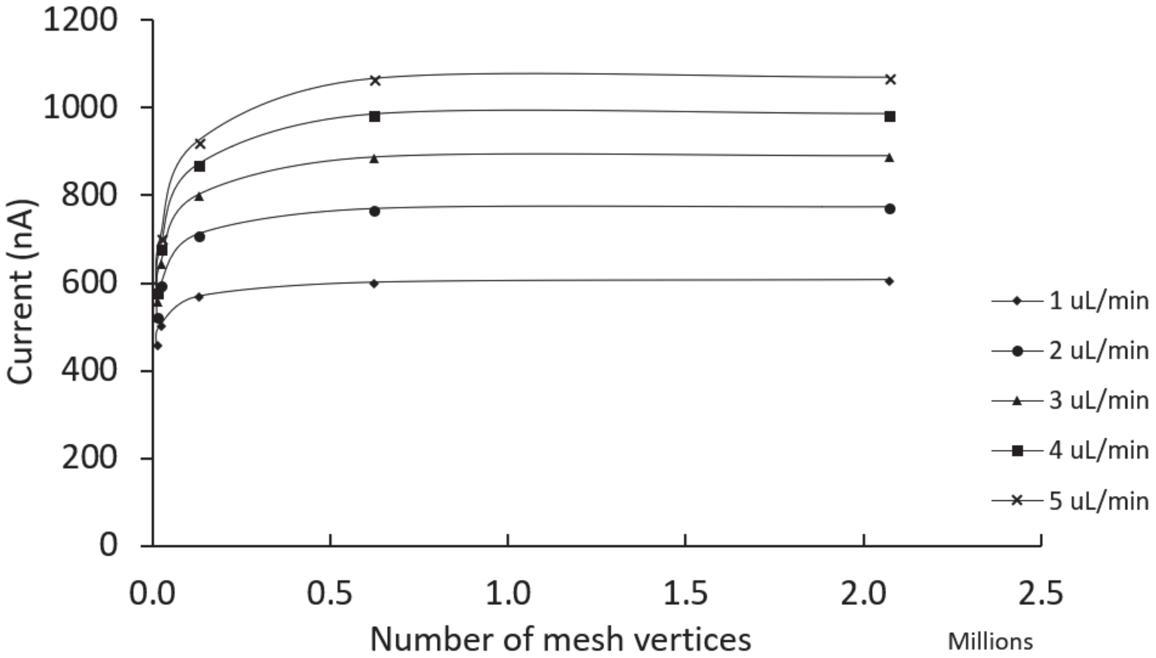

Figure S5. Predicted limiting current at different flow rates using COMSOL, calculated at different mesh densities. It shows asymptotic convergence to a limiting current value as we increase the mesh density.

Table S1. Meshing sequence and properties for a microdisk channel electrode at different mesh densities.

**Node:**						
**Mesh1**	**Description**					
Size	Calibrate for: fluid dynamics					
	Predefined for:	fine	fine	fine	coarse	coarser
**Edge1**						
Size1	Calibrate for: general physics					
	Edge: electrode (9-12)					
	Custom. Max element size (m)	2.50E-07	5.00E-07	2.00E-06	4.00E-06	5.00E-06
**Free Triangular 1**						
Size 1	Boundary: top wall (6)					
	Custom. Max element size (m)	5.00E-06	5.00E-06	5.00E-06	1.20E-05	5.00E-05
	Custom. Min element size (m)	1.00E-07	1.00E-07	1.00E-07	1.00E-07	1.00E-07
	Custom. Max element growth rate	1.025	1.025	1.025	1.025	1.025
Size 2	Boundary: electrode (4)					
	Custom. Max element size (m)	5.00E-07	1.00E-06	5.00E-06	1.20E-05	2.00E-05
	Custom. Min element size (m)	2.50E-08	2.50E-08	2.50E-08	2.50E-08	2.50E-08
	Custom. Max element growth rate	1.025	1.025	1.025	1.025	1.025
**Corner Refinement1**	Channel walls (2-5)	auto settings				
**Free Tetrahedral 1**	Remaining	auto settings				
**Boundary Layers 1**						
Boundary Layer Properties	# of boundary layers:6					
	boundary layer stretching factor: 1.2					
	first layer thickness (m): 5E-7					
DOF		12419868	3710472	758802	127434	50960
Internal DOF		384265	134153	35453	11115	3272
Mesh vertices		2069978	618412	126267	21239	10192
Tetrahedra		8531627	2683275	643003	93610	41017
Pyramids		7548	3768	960	480	384
Prisms		1111144	277692	21492	5760	3336
Triangles		380068	131332	33808	10230	5874
Edge elements		2092	1404	816	436	328
Skewness		0.6723	0.6692	0.6632	0.6606	0.6849

Table S2. Meshing sequence and properties for a modified geometry model for channel disk microelectrode.

Node:			
**Mesh1**	**Description**	**Trapezoid and recess**	**Trapezoid**
Size	Calibrate for: fluid dynamics		
	Predefined for:	fine	fine
**Edge1**			
Size1	Calibrate for: general physics		
	Edge: electrode (33-34, 40, 43)		
	Custom. Max element size (m)	2.00E-07	2.50E-07
**Free Triangular 1**			
Size 1	Boundary: top wall (6)		
	Custom. Max element size (m)	1.00E-05	1.00E-05
	Custom. Min element size (m)	1.00E-07	1.00E-07
	Custom. Max element growth rate	1.05	1.05
Size 2	Boundary: electrode (4)		
	Custom. Max element size (m)	2.00E-07	1.00E-06
	Custom. Min element size (m)	2.50E-08	2.50E-08
	Custom. Max element growth rate	1.025	1.025
**Corner Refinement1**	Channel walls (2-5)	auto settings	
**Free Tetrahedral 1**	Remaining	auto settings	
**Boundary Layers 1**			
Boundary Layer Properties 1	Boundary: electrode (4)		
	# of boundary layers: 6		
	boundary layer stretching factor: 1.2		
	first layer thickness (m): 5E-7		
DOF		6930176	11189923
Internal DOF		465529	798381
Mesh vertices		590438	953192
Tetrahedra		2710466	5340521
Pyramids		13038	
Prisms		190366	
Triangles		192536	324682
Edge elements		5658	7656
Skewness		0.6636	0.6383

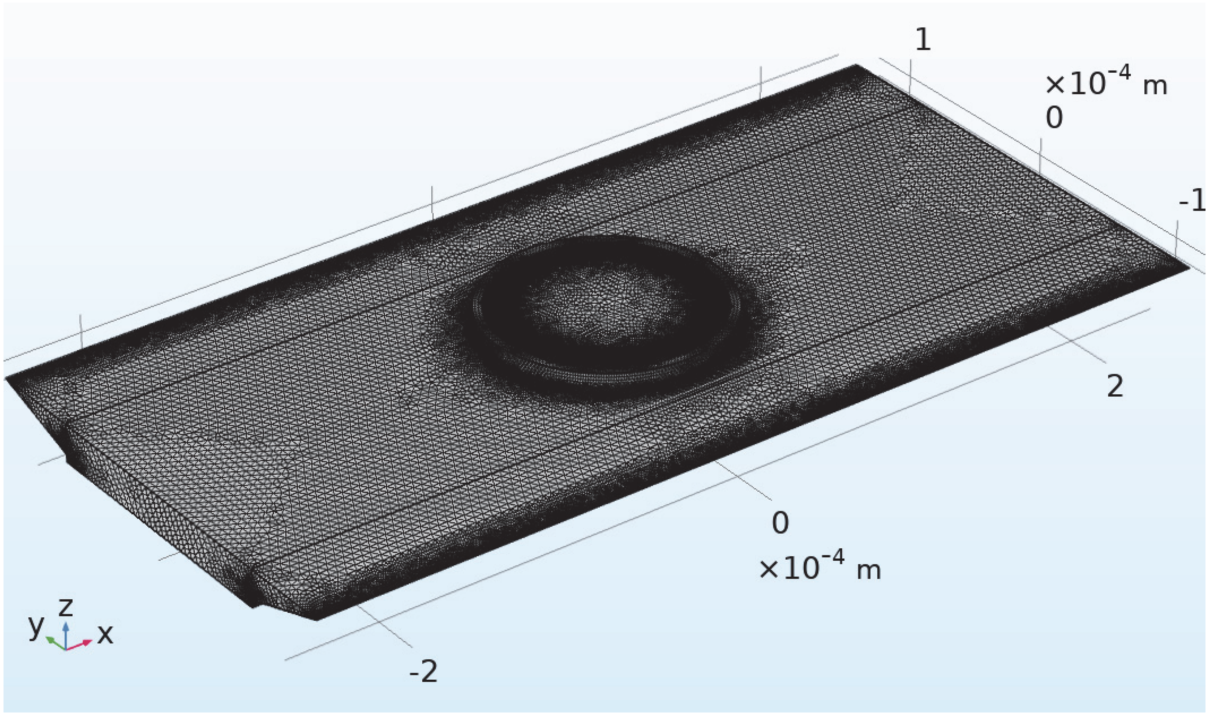

Figure S6. Meshing of channel microelectrode channel trapezoidal shape and recessed electrode, showing higher density at electrode border and channel top outer edges.

4. Band electrode model and Levich equation comparison

The band channel electrode was modeled in 3D in COMSOL in order to compare it against the Levich equation[3] which is an analytical expression used to calculate the mass transfer-limited current. The Levich equation approximates the band electrode by neglecting the transversal and axial diffusion and approximating the parabolic velocity profile with a linear profile. Table S4 shows the parameters used for the band electrode simulation. Additionally, diffusion in the *x* and *y* directions have been disabled along the flow direction and transversally, by setting the diffusion coefficient in those directions equal to zero. Figure S7 shows the model mesh for the band electrode simulation at ~368k nodes.

Table S5 summarizes the mesh sequence properties for the COMSOL band electrode at two elevated mesh densities of about 368k and 765k nodes. In Figure S8 we demonstrate that, already at ~368k nodes, the COMSOL model approximates the Levich equation very closely (~1.3% difference). This means that COMSOL simulations in 3D can approximate the Levich equation results closely at these mesh densities. COMSOL in 3D then becomes a useful tool for other systems where the geometry and assumptions of the Levich equation do not apply, like the case of the disk channel electrode.

Table S3. Parameters for band electrode simulation in COMSOL.

**Parameter**	**Value**	**Description**
w_electrode	54 [µm]	electrode length
w_channel	270 [µm]	Channel and electrode width
h_channel	528[µm]	Channel height
l_channel	2*h_channel	Channel length
f_in	15-90 uL/min	Flow in
A	w_channel*h_channel	Area perp to flow
c_bulk	1.07[mmol/L]	Reactant concentration
Dr	2.3e-5[cm^2/s]	Product diffusion coefficient
v_in	f_in/A	velocity in
T0	293.15[K]	Temperature
vmax	f_in/A*3/2	Max velocity
v	f_in/A	Avg velocity

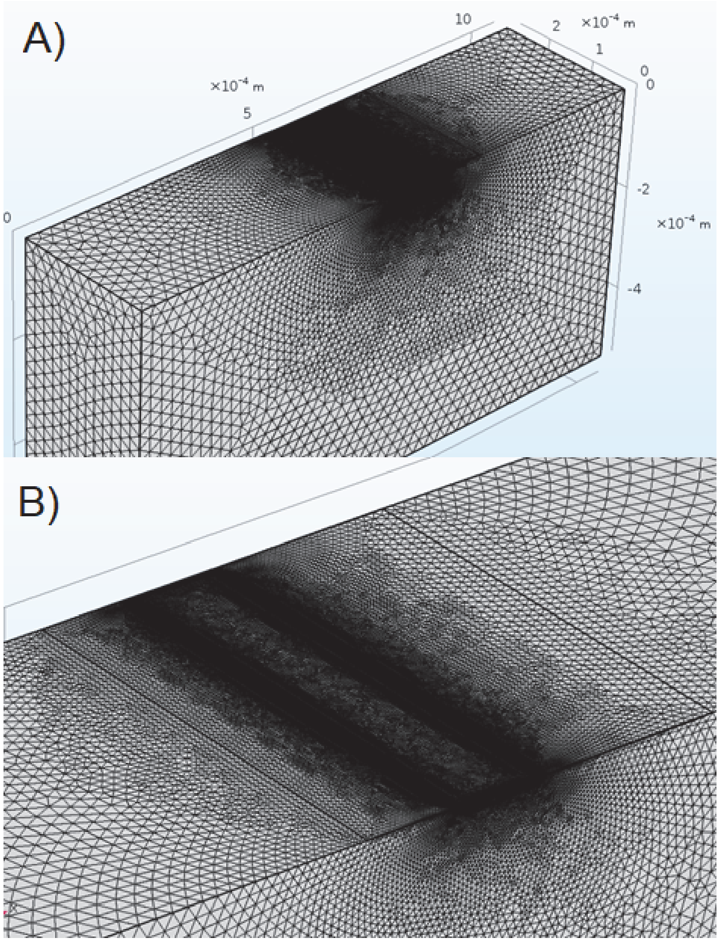

Figure S7. (A) Band channel electrode model meshing for comparison with Levich equation, at 368k mesh vertices. (B) Zoom on the electrode mesh, showing a higher density at the border of the border of the electrode.

Table S4. Meshing sequence and properties for a band channel electrode at two different mesh densities.

**Node:**			
**Mesh1**	**Description**		
Size	Calibrate for: fluid dynamics		
	Predefined for:	Normal	Normal
Size 1	Boundary: channel walls (2-9)		
	Custom. Max element size (m)	2.00E-04	2.00E-04
	Custom. Min element size (m)	5.00E-07	5.00E-07
	Custom. Max element growth rate	1.05	1.05
**Edge1**			
Size1	Calibrate for: general physics		
	Edge: electrode (12-15)		
	Custom. Max element size (m)	5.00E-07	1.00E-06
	Custom. Min element size (m)	1.00E-07	1.00E-07
	Custom. Max element growth rate	1.01	1.01
**Free Triangular 1**			
Size 1	Boundary: infront and after electrode (6,8)		
	Custom. Max element size (m)	5.00E-06	5.00E-06
	Custom. Min element size (m)	3.00E-07	3.00E-07
Size 2	Boundary: electrode (7)		
	Custom. Max element size (m)	2.00E-06	5.00E-06
	Custom. Min element size (m)	3.00E-07	3.00E-07
	Custom. Max element growth rate	1.01	1.01
**Corner Refinement1**	Channel walls (2-5)	auto settings	
**Free Tetrahedral 1**	Remaining	auto settings	
**Boundary Layers 1**			
Boundary Layer Properties 1	Boundary: electrode and sourrounding (6-8)		
	# of boundary layers:4		
	boundary layer stretching factor: 1.2		
	first layer thickness (m): 1E-7		
DOF		5954465	2858280
Internal DOF		7279236	3593316
Mesh vertices		765100	368110
Tetrahedra		3032220	1516265
Pyramids		1172	912
Prisms		436920	186000
Triangles		171180	89256
Edge elements		2416	1531
Skewness		0.6776	0.6784

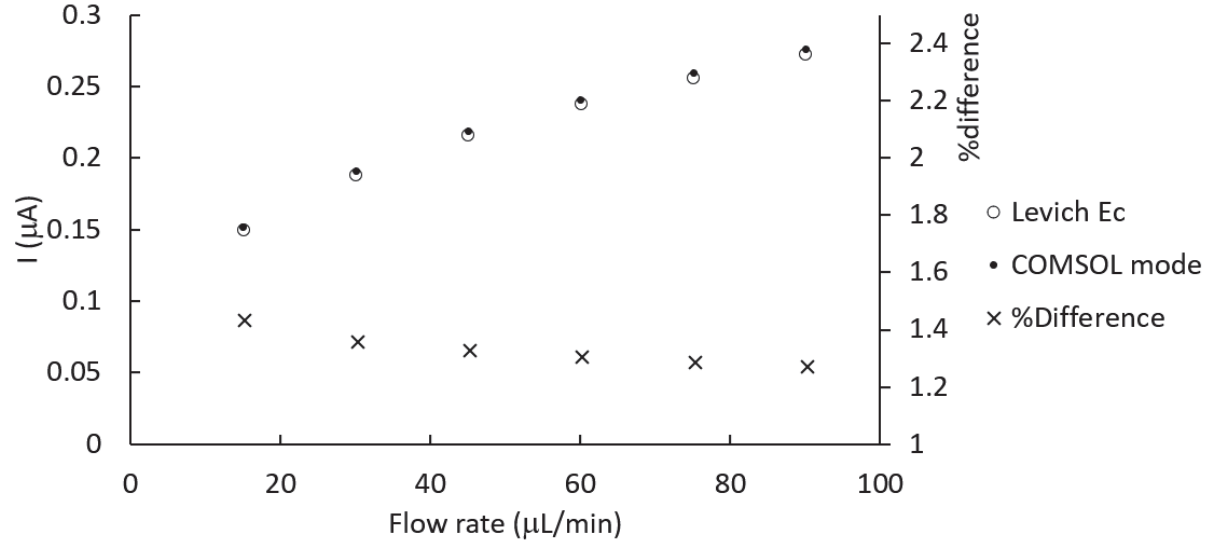

Figure S8. Predicted limiting current at different flow rates 15-90 μL/min for microband channel electrode on the left vertical axis, using the Levich equation and the COMSOL model. The percentage difference of the two predicted currents is shown on the right vertical axis and demonstrates a good agreement with differences below 1.4%.

5. Nernst-Plank Equation Model

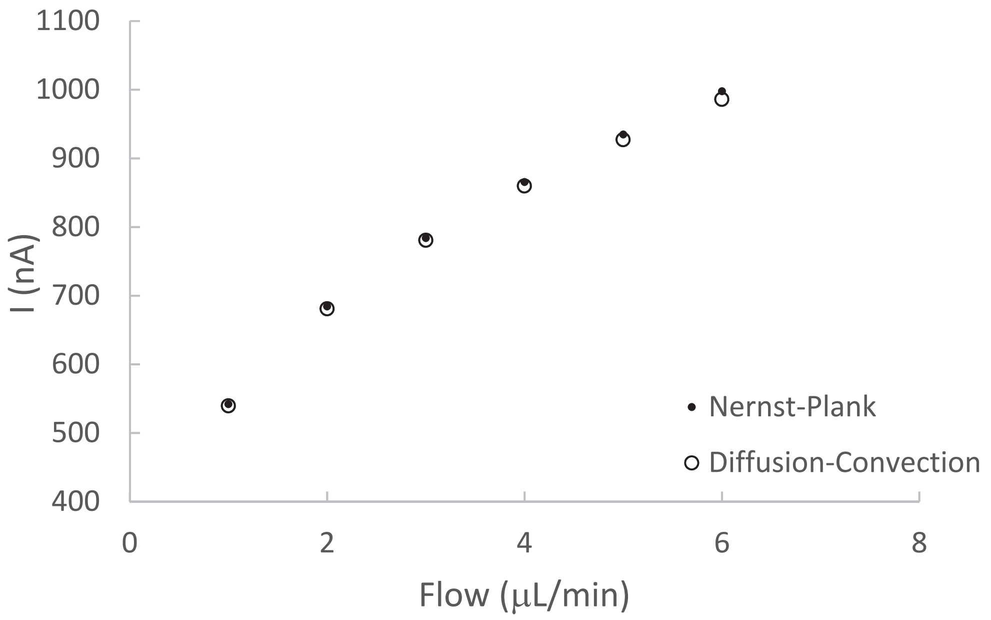

Figure S9. Predicted limiting currents at different flow rates using the complete Nernst-Plank Equation[3] which considers ion migration and the effect of background electrolyte. The electrode currents obtained with this model are close within ~1% to the ones obtained with the simpler diffusion-convection model, also shown in this figure. The geometry used is shown in Figure 8, with a 5 μm electrode recess.

6. Concluding remarks

Channel profilometry and electrode SEM imaging allowed us to include more realistic geometries in COMSOL 3D finite element simulations of a channel disc electrode and significantly improved agreement between the limiting currents predicted by the simulation and those measured experimentally. We also found that the value of the simulated limiting current in COMSOL depends heavily on the model mesh density, especially around the electrode edges where higher current densities lead to steep concentration gradients. It's necessary to check for convergence of the simulated current as we increase the mesh density. We also confirmed that COMSOL was able to predict the mass transfer-limited current of a band channel electrode in 3D, differing by less than 1.4% from the analytical solution derived from the well-known Levich equation.[3] The diffusion-convection model employed in the main paper is a simplification of the Nernst-Plank equation when we don't consider ion migration due to excess background electrolyte. The currents resulting in this model give similar results as the simplified version within ~1% difference.

7. References

1. I. J. Cutress, E. J. Dickinson, and R. G. Compton, *Journal of Electroanalytical Chemistry,* **638** (1), 76-83 (2010).
2. H. L. Woodvine, J. G. Terry, A. J. Walton, and A. R. Mount, *Analyst,* **135** (5), 1058-1065 (2010).
3. R. G. Compton and C. E. Banks, *Understanding voltammetry*, Imperial Collegue Press, London (2011).
